# Structural Basis for Cytochrome *c* Y67H Mutant to Function as a Peroxidase

**DOI:** 10.1371/journal.pone.0107305

**Published:** 2014-09-11

**Authors:** Wenxian Lan, Zhonghua Wang, Zhongzheng Yang, Tianlei Ying, Xu Zhang, Xiangshi Tan, Maili Liu, Chunyang Cao, Zhong-Xian Huang

**Affiliations:** 1 State Key Laboratory of Natural Products and Bioorganic Chemistry, Shanghai Institute of Organic Chemistry, Chinese Academy of Sciences, Shanghai, China; 2 Chemical Biology Laboratory, Department of Chemistry, Fudan University, Shanghai, P. R. China; 3 State Key Laboratory of Magnetic Resonance and Atomic and Molecular Physics, Wuhan Institute of Physics and Mathematics, Chinese Academy of Sciences, Wuhan, China; Università di Napoli Federico II, Italy

## Abstract

The catalytic activity of cytochrome *c* (cyt *c*) to peroxidize cardiolipin to its oxidized form is required for the release of pro-apoptotic factors from mitochondria, and for execution of the subsequent apoptotic steps. However, the structural basis for this peroxidation reaction remains unclear. In this paper, we determined the three-dimensional NMR solution structure of yeast cyt *c* Y67H variant with high peroxidase activity, which is almost similar to that of its native form. The structure reveals that the hydrogen bond between Met80 and residue 67 is disrupted. This change destabilizes the sixth coordination bond between heme Fe^3+^ ion and Met80 sulfur atom in the Y67H variant, and further makes it more easily be broken at low pH conditions. The steady-state studies indicate that the Y67H variant has the highest peroxidase activities when pH condition is between 4.0 and 5.2. Finally, a mechanism is suggested for the peroxidation of cardiolipin catalyzed by the Y67H variant, where the residue His67 acts as a distal histidine, its protonation facilitates O-O bond cleavage of H_2_O_2_ by functioning as an acidic catalyst.

## Introduction

Recently, it has been proposed that proteins can inherently possess a variety of conformations *in vivo*, and hence have functional diversity [1]. However, it's difficult to understand the mechanisms involved in different conformers from native protein to “non-native” protein [2]–[3], because it's hard to obtain the single conformer of the protein at different states. Cytochrome *c* (*i.e.*, cyt *c*), a well-known electron transfer hemoprotein, has several conformers to carry out its biological functions beyond respiration [4]. Two of them have given rise to special attentions. One is the so-called “alkaline conformer” with weak peroxidase activity [5]–[6], in which the axial ligand of the heme iron, Met80, is replaced by Lys72, Lys73 or Lys79 at alkaline pH condition. The structural investigation on the alkaline conformer implied that this conformer might work as an electron transfer gate, and a folding intermediate [7]. The other is the “pro-apoptotic” conformer with enhanced peroxidase activity [8]–[10]. It can catalyze peroxidation of cardiolipin (a mitochondria specific phospholid) to its oxidized form, which is essential for the release of pro-apoptotic factors from mitochondria, and for performing the subsequent apoptotic processes. But the structural basis of this catalytic peroxidation remains unclear.

**Figure 1:**
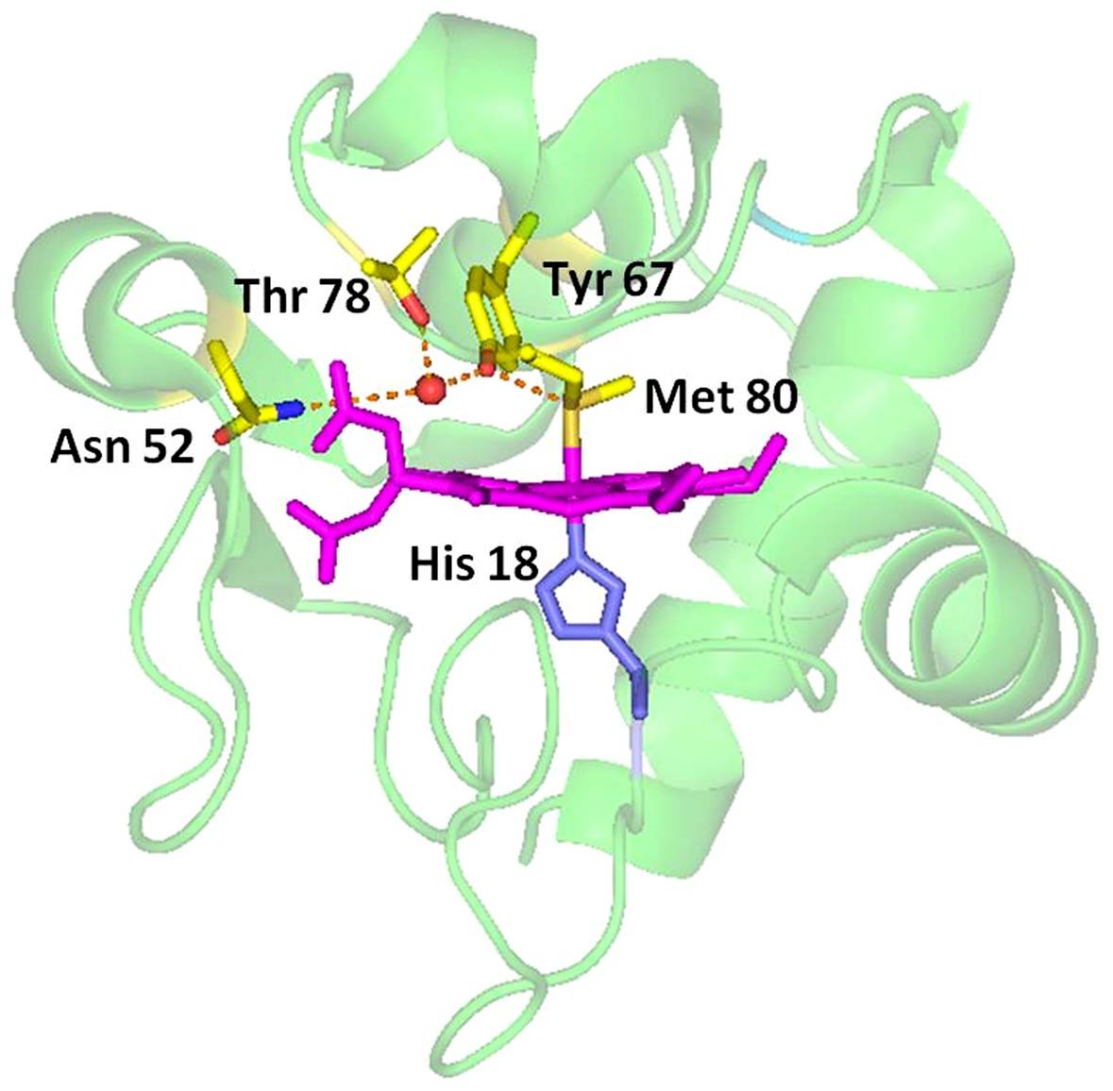
Structure of the heme region of cyt *c* and the location of Tyr67 in the structure (pdb code: 1YIC), showing the hydrogen bond network formed by amino acids His18, Tyr67, Met80, Asn52 and Thr78.

In cyt *c*, Tyr67 is a highly conserved residue except that in the mitochondrial cyt *c*-558 of *Euglena gracilis*, where the residue 67 is phenylalanine [11]. The residue Tyr67 is extremely important in the electron transfer because its side-chain extends into the heme pocket and forms a hydrogen bond network with axial ligand Met80, conserved residues Asn52 and Thr78 and a buried water molecule identified in the X-ray structure of cyt *c* (pdb code 2YCC) [12]–[13], as shown in Figure 1. This hydrogen bond network not only helps to maintain the sulfur atom of Met80 in its heme iron ligand position and thus controls the redox potential of the protein, but also modulates the flexibility of the nearby peptide chain segment (*i.e.*, residues 65 to 72), therefore affects chemical properties of the protein. More interestingly, Tyr67 is located at the distal position of heme pocket, the distance between Tyr67 –OH group and heme Fe^3+^ is 4.3 Å, which is close to the distances of 5.84 Å and 5.55 Å between Nε2 atom of the distal histidine and heme iron ion in horseradish peroxidase (*i.e.*, HRP, pdb code 1H5A) and cytochrome *c* peroxidase (*i.e.*, C*c*P, pdb code 2CYP) [14]–[15], respectively. Thus, Tyr67 was suggested to be a possible apoptotic trigger via a conformation resulted by site-directed mutagenesis [16]–[17].

Originally, in order to convert cyt *c* into a peroxidase, we successfully designed cyt *c* Y67H and Y67R variants [16]–[17]. Replacement of Tyr67 by His67 is to introduce a distal histidine, while mutation from Tyr67 to Arg67 is to produce a distal arginine. The results from electronic spectrum (a weak charge transfer band centered at 695 nm for Fe-S bond) and CD spectrum (loss of a negative band centered at 416 nm) implied that the heme Fe-S (Met80) bond was not broken in the Y67H variant, but really ruptured in the Y67R variant. Moreover, the midpoint temperatures (*T*_m_) of the Y67H variant (50°C) and of the Y67R variant (30°C) are much lower than those of the wild-type cyt *c* (higher than 80°C) and of its Y67F variant (90°C) [18], indicating that the detachment of Met80 to heme iron is significantly easier in the Y67H variant than that in wild-type cyt *c*. Both Y67H and Y67R variants have high peroxidase activities [16]–[17]. Thus, the Y67H and Y67R mutants stand for a typical pro-apoptotic conformer of cyt *c*. Since there is no solid structural evidences to interpret the basis of peroxidation in this state, it's necessary to study their three dimensional conformation. Thus, in this paper, by conventional two-dimensional ^1^H-^1^H NMR methods [19], we determined the NMR solution structure of the cyt *c* Y67H variant.

In this paper we investigate the structure of the Y67H mutant. This mutant was constructed using the pBTR2 plasmid which encodes the yeast iso-1-cyt *c* (CYC1) genes. This is a cytochrome c variant carrying two point mutations K72A/C102T. The change from Cys102 to Thr102 is used to prevent disulfide dimerization of the protein and protein auto-reduction [7], [20]–[21], which may alter the cytochrome's thermodynamic and spectroscopic parameters. The mutation from Lys72 to Ala72 is utilized to prevent this residue from serving as a ligand in the alkaline form of cyt *c* at pH 7.0. For the sake of simplicity, throughout this paper, the double-site K72A/C102T variant is used as the reference and referred to as native yeast iso-1 cyt *c*. The triple mutants Y67H/K72A/C102T and Y67R/K72A/C102T used in this study are referred to the Y67H and Y67R variants, respectively.

## Results

### Heme destruction upon native cyt *c* and its Y67H variant reacting with H_2_O_2_

**Figure 2:**
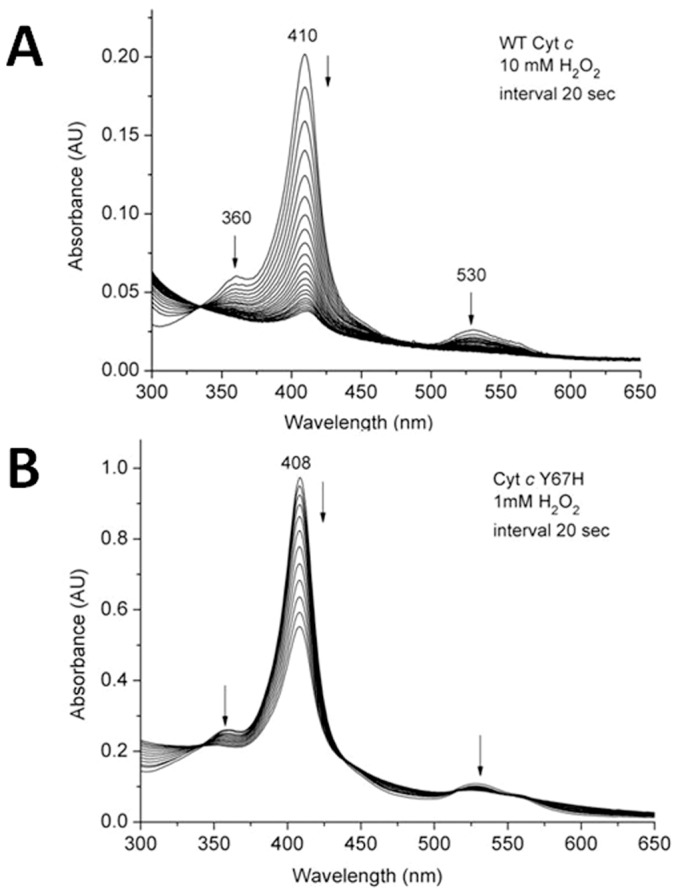
Heme destruction of the wild-type cyt *c* (A) and its Y67H variant (B) as indicated by dissipation of the Soret band. Spectra were scanned every 20 sec, and the arrows indicate the direction of change. The concentrations of H_2_O_2_ were 10 mM in (A) and 1 mM in (B), the pH condition in both cases is 6.0.

**Figure 3:**
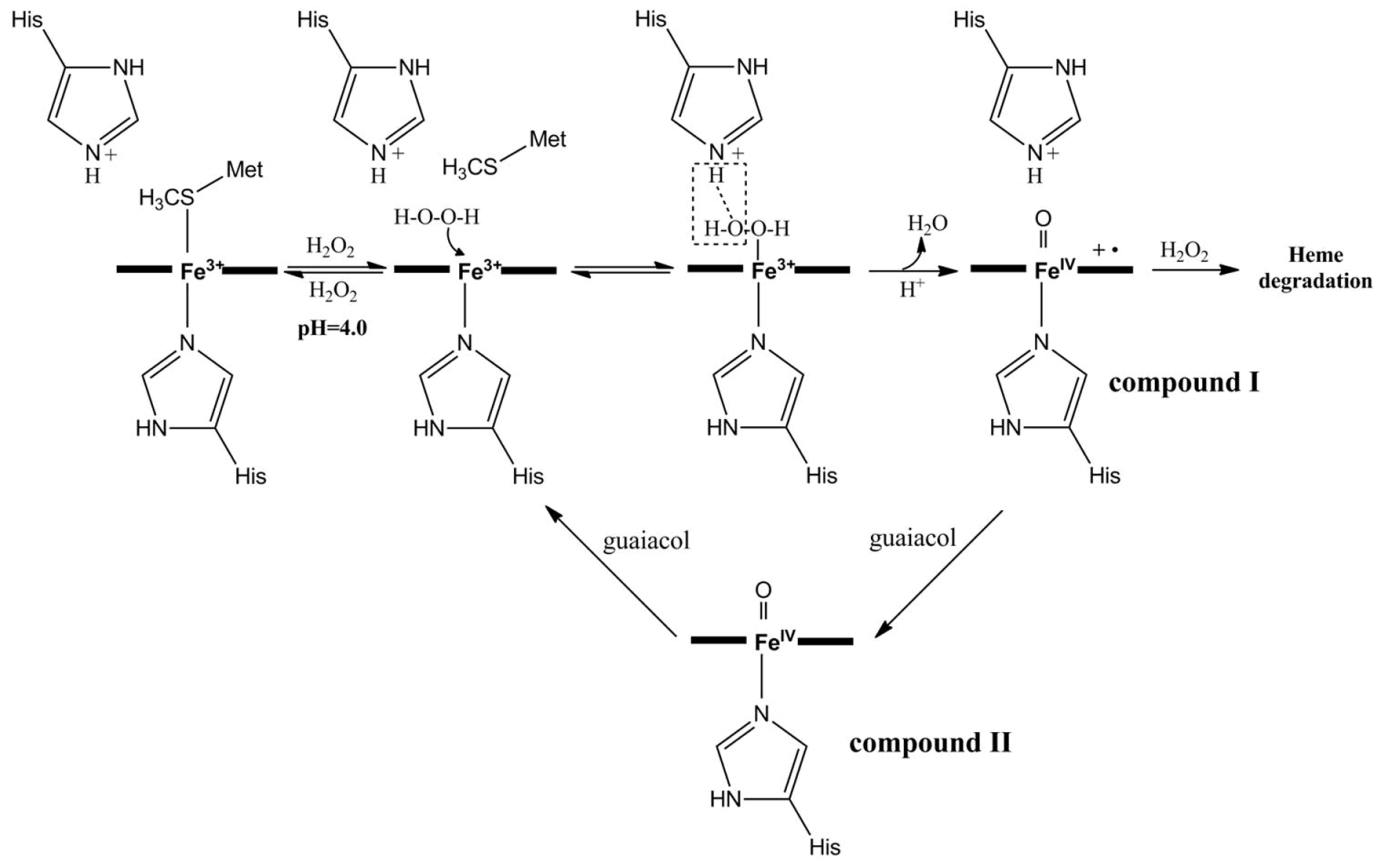
The possible mechanism for guaiacol reaction with H_2_O_2_ catalyzed by the cyt *c* Y67H mutant at pH 4.0.

**Figure 4:**
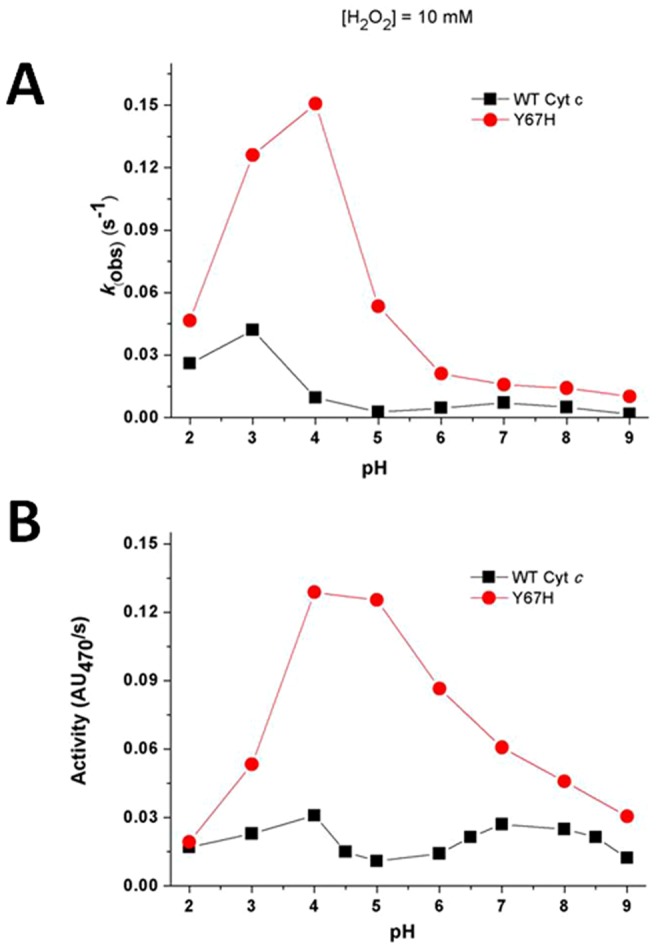
Steady-state kinetics studies on (A) heme destruction and (B) peroxidase activity vs pH change of wild-type cyt *c* and its Y67H mutant.

To better understand the mechanism of peroxidation by the Y67H variant, we first probed the stability of native cyt *c* and its Y67H variant only in the presence of H_2_O_2_. As shown in Figure 2 (For clear description, the concentration of H_2_O_2_ in Figure 2B is 1 mM, because the absorption at 410 nm decays extremely fast when it is 10 mM), in the absence of guaiacol, once H_2_O_2_ reacts with native cyt *c* and its Y67H mutant, the visible absorption maximal values at ∼408 nm (Soret band), 530 nm (α+β bands) and ∼353 nm (δ band) become weaker and weaker, indicating that the heme ring in both proteins is destructed very quickly [22]. This observation is apparently different from myoglobin [22]–[23], which in the absence of substrate for peroxidation reacts with H_2_O_2_ to generate compound II as confirmed by the absorption band at ∼420 nm (compound II structure is shown in Figure 3). Figure 4A demonstrates that the native cyt *c* and its Y67H mutant have different behaviors vs pH variation. For the Y67H variant, the observed rate (*k*_obs_) of heme destruction is the highest at about pH 4.0. When pH value is equal to or higher than 6.0, the *k*_obs_ values for the Y67H variant are almost kept at 0.02 s^−1^, but is dramatically decreased when pH is less than 4.0. For the native cyt *c*, the *k*_obs_ value of heme destruction is not completely changed upon pH condition being varied. Our previous acid titration studies on both native cyt *c* and its Y67H variant [17] implied that, different from wild-type cyt *c*, at pH 4.0–5.2 (especially at pH 4.0), Fe-S bond was clearly cleaved since the absorbance at 695 nm underwent a sharp decrease. The cleavage of Fe-S bond generates a vacancy for H_2_O_2_ coordination, so that heme ring is easier to be oxidized by H_2_O_2_. Thus, the *k*_obs_ value of heme destruction of the Y67H variant at pH between 4.0 and 5.2 is significantly higher than those at other pH conditions.

### Steady-state kinetic studies

After we performed heme destruction by H_2_O_2_, we then investigated the peroxidase activity of wild-type cyt *c* and its Y67H mutant on guaiacol vs pH change. As shown in Figure 4B, upon pH being varied, the native cyt *c* displays no significant changes in the peroxidase activity, but the Y67H mutant demonstrates the highest peroxidase activity when pH is between 4.0 and 5.2, increased by about 4–10 folds, compared to that of native cyt *c*. As we know, at pH 4.0–5.2 the axial Fe-S bond is cleaved, generating a channel for loading H_2_O_2_ and substrate guaiacol which enhances the peroxidation reaction.

### Sequence-specific assignment

Using two dimensional (2D) ^1^H-^1^H total correlation spectroscopy (TOCSY) and correlation spectroscopy (COSY) in H_2_O and D_2_O for spin patterns and 2D ^1^H-^1^H nuclear Overhauser effect spectroscopy (NOESY) spectra in H_2_O for sequential NH-NH and Hα-HN connectivities, we made the assignments for the Y67H mutant under our experimental conditions (20°C and 50 mM phosphate buffer, pH 7.0), according to the extensive lists of assignments for yeast cyt *c* reported in the literatures [24]–[26]. The chemical shifts of residues Gln16, Thr19, Gly29, Asn31 and Leu32 were firstly identified because their spin patterns were resolved outside the diamagnetic envelope. The sequence-specific assignment was successfully performed in the regions of residues 3–13, 50–54, 61–68, 71–74 and 88–102, referencing with the data for the native cyt *c* [24]–[26]. Residues, especially close to the heme ring and the mutation site, exhibit different chemical shifts, which were finally assigned and given in Table 1. In total, more than 88% of the expected proton resonances had been assigned for the Y67H mutant.

**Table 1:** New assignments of Y67H mutant.

residues	chemical shift (ppm)
His^18^	HN 11.10, Hα 8.84, H*β*1 8.36, H*β*2 15.55, H*δ*1 11.81, H*δ*2 22.65, H*ε*1 −23.85
Pro^30^	H*α* 4.12, H*γ*1 −0.71, H*γ*2 0.01, H*δ* −1.61
Leu^32^	*δ*1-CH_3_ 0.62, *δ*2-CH_3_ 1.08
Ile^35^	*γ*-CH_3_ −0.12, *δ*-CH_3_ 0.23, *γ*-CH_2_ 0.49, 1.03
Trp^59^	H*ε* 7.52
His^67^	HN 8.30, Hα 4.28, H*β*1 3.12, H*β*2 3.18, H*δ* 6.82, H*ε*2 8.00
Leu^68^	H*α* 3.36, H*β*1 0.23, H*β*2 1.05, H*γ* 0.68, *δ*1-CH_3_ −3.05, *δ*2-CH_3_ −1.02
Met^80^	HN 8.67, Hγ1 −27.8, ε-CH_3_ −19.5
Phe^82^	HN 8.44, H*α* 4.56, H*β*1 2.98, H*β*2 3.25
Leu^85^	HN 8.16, H*α* 4.25, H*β*1 0.72, H*β*2 1.13, H*γ* 0.83, *δ*1-CH_3_ 0.19, *δ*2-CH_3_ 0.54

### NMR signals assignment of axial ligands and heme of the Y67H variant

**Figure 5:**
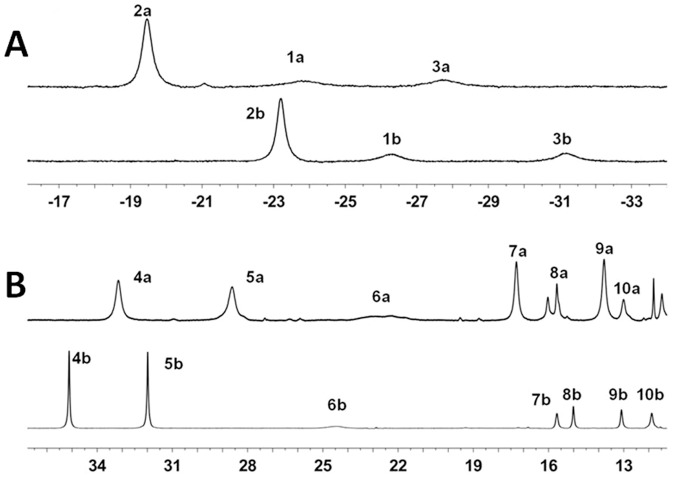
1D ^1^H-NMR spectra of cyt *c*. (A) The high-field shifted region of the native cyt c (down) and its Y67H variant (upper) spectra. (B) The down-field shifted region of the native cyt *c* (down) and its Y67H variant (upper) spectra. The peaks in 1D ^1^H-NMR spectrum were assigned as (1a) His18 Hε1, (2a) His71 Hε1, (3a) His71 Hδ2, (4a) heme 8-CH_3_, (5a) His18 Hδ2, (6a) heme 3-CH_3_, (7a) heme 5-CH_3_, (8a) His18 Hβ2, (9a) heme 1-CH_3_ and (10a) heme 7-Hα2, respectively. The peaks in 1D ^1^H-NMR spectrum of native cyt *c* were assigned as (1b) His18 Hε1, (2b) Met80 ε-CH_3_, (3b) Met80 Hγ, (4b) heme 8-CH_3_, (5b) heme 3-CH_3_, (6b) His18 Hδ2, (7b) heme 7-Hα2, (8b) His18 Hβ2, (9b) heme 7-Hα1 and (10b) His18 Hδ1, respectively.

One dimensional (1D) ^1^H NMR spectra shown in Figure 5 indicate that the hyperfine shifts of the Y67H mutant are completely distinct from those of the native cyt *c*, so NMR signals of heme of the Y67H variant cannot be assigned directly according to the similarity of the 1D ^1^H-NMR spectra between the native cyt *c* and its Y67H mutant. The complete assignments of hyperfine shifted signals of the heme protons, the axial ligands His18 and Met80, and the heme bound Cys14 and Cys17 of Y67H were achieved through analysis of the 2D ^1^H-^1^H NOESY spectra tailored to the relaxation properties of these paramagnetic resonances and through one-dimensional nuclear Overhauser effect (NOE) experiments, as reported before [24]–[25]. The characteristic peaks at −31.0 ppm (peak 3b) and −22.7 ppm (peak 2b) in the high-field shifted region of 1D ^1^H-NMR spectrum of native cyt *c* (Figure 5) were previously assigned as the axial ligand Met80 H*γ*1 and *ε*-CH_3_ [24]–[25], respectively. Their corresponding peaks are at −27.8 ppm (peak 3a) and −19.5 ppm (peak 2a) in the high-field shifted region of 1D ^1^H-NMR spectrum of the Y67H mutant. Thus, NMR data suggest that the Met80 is still served as the sixth axial ligand in Y67H mutant.

The well resolved signals at 33.1, 14.0, 28.4, and 17.7 ppm (Figure 5B) were suggested to be the four methyl groups of the heme of Y67H mutant. The signal at 14.0 ppm can be assigned to 1-CH_3_ because it has NOEs with the signals of a thioether group and methyl groups of Leu32, Leu68 and Leu94, which were also observed in native cyt *c*. The resonances at 33.1 and 14.0 ppm display strong NOE connectivities to a signal at −0.3 ppm. The only proton which is equidistant from two methyl groups is the meso-Hδ, positioned between the 1-CH_3_ and the 8-CH_3_ groups. The assignment of the signal at 33.1 ppm as 8-CH_3_ is consistent with its connectivities with *δ*_2_-CH_3_ of Leu32 and ε-CH_3_ of Met64. The remaining resonances are assigned as 3-CH_3_ and 5-CH_3_. The resonance at 17.7 ppm was assigned as 5-CH_3_ due to its NOE connectivities to Gly29 Hα (at −0.5 ppm) and Pro30 Hδ (at −1.6 ppm). The resonances of the other heme substituents were assigned by analyzing the NOESY connectivities, starting from position 1 and 3 and “hopping” around the macrocycle (Table 2 and Figure S1 in File S1).

**Table 2:** The assignment of heme protons of cyt *c* Y67H mutant.

atoms	cyt *c* Y67H (ppm)
1-CH_3_	14.0
2-CH	1.47
2-CH_3_	−1.25
meso- H*_α_*	3.96
3-CH_3_	28.4
4-CH	3.02
4-CH_3_	2.38
meso- H*_β_*	−0.39
5-CH_3_	17.7
6- H*α*1	−1.05
6- H*α*2	2.42
6- H*β*1	1.05
6- H*β*2	2.35
meso- H*_γ_*	7.71
7- H*α*1	11.09
7- H*α*2	13.18
7- H*β*1	0.51
7- H*β*2	1.30
8-CH_3_	33.1
meso- H*_δ_*	−0.31

The peaks at the positions of −26.32 ppm (peak 1b) and 24.54 ppm (peak 6b) (Figure 5) which belong to the side-chain aromatic protons H*ε*1 and H*δ*2 of the axial ligand His18 in native cyt *c* [24]–[25], respectively, are shifted to the positions (peak 1a, −23.85 ppm; peak 6a, 22.65 ppm) in 1D ^1^H-NMR spectra of the Y67H mutant (Figure 5). The saturation of the signal at −23.85 ppm allows the detection of a NOE with a proton resonating at 11.10 ppm, which displays NOE connectivities to two H*β* protons of His18 resonating at 15.55 ppm and 8.36 ppm. The assignments of two H*β* protons of His18 are consistent with the NOE patterns between them and the amide HN protons of Leu32 and Thr19 observed in native cyt *c* [24]–[25]. So the signals at 11.81 ppm and −23.85 ppm were assigned as the side chain atoms H*δ*1 and H*ε*1 of His18, respectively. The detectability of the signal H*δ*1 of His18 indicates that its H-bond with Pro30 is preserved in the Y67H mutant. The NOE patterns between the two H*β* protons of His18 and the signal at 22.65 ppm imply that this signal is from H*δ*2 of His18, which also displays a NOE connectivity with a thioether group at 2-position (1.47 ppm) in the heme ring. The saturation of the signal at 11.10 ppm indicates that it has NOE patterns with two H*β* protons of His18, suggesting that it comes from the backbone NH of His18, which is further confirmed by the NOE peaks between it and the H*α* of Cys17 and Thr19 HN. The assignment of H*α* of His18 at 8.84 ppm was performed based on NOE patterns between this signal and the HN proton, H*β*2 proton of His18 and between this signal and HN of Thr19. The 1D ^1^H-NMR spectra of the Y67H mutant show broad signals at −19.5 ppm (peak 2a in Figure 5A) and 22.65 ppm (peak 6a in Figure 5B), both do not exchange with deuterium in D_2_O solution. The line widths of these signals suggest that they are from protons of the metal ligands, most probably the axial ligands. Thus, based on the assignments of native cyt *c* [24]–[26], these were appointed as Met80 *ε*-CH_3_ and His18 H*δ*2, respectively.

### NMR solution structure determination of the Y67H mutant

The helical conformations of the Y67H mutant were characterized by using the reported techniques [24]–[26], further ensured by using observed NOE patterns (Figure S2 in File S1), are displayed in the regions of 3–13, 50–54, 61–68, 71–74, and 88–102 aa. They are almost similar to those presented in the solution structures of the wild-type *Saccharomyces cerevisiae* iso-1 cyt *c* [24]–[26]. The helix IV (71–74 aa) in native cyt *c* is disappeared in some conformers of the Y67H mutant.

**Figure 6:**
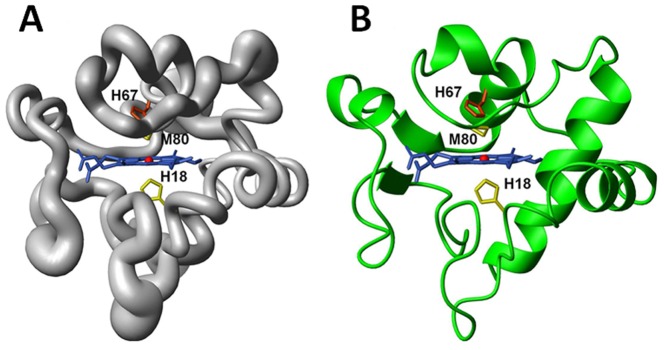
3D solution structures of cyt *c* Y67H variant. (A) Backbone of the family of 20 Y67H structures shown as a tube of variable radius. The radius is proportional to the RMSD of each residue. (B) Ribbon diagram representation of the Y67H mutant. The figures are generated with the program MOLMOL [48]. The iron ion was represented as ball in red.

The solution structures of the Y67H variant were determined by using meaningful and acceptable 1699 NOEs through the program XPLOR [27]. The heme group, the axial ligands, and the two cysteine (Cys14 and Cys17) covalently attached to the porphyrin are treated as new patch residues. Among them, the patch residue HEC stands for cyt *c*-type heme ring, PHEM is served as one axial ligand histidine of heme iron, and PHMT represents methionine worked as another axial ligand of heme iron. PHCB and PHCC are used as the residues Cys14 and Cys17. These applications for solution structure calculation of cyt *c*-type heme contained proteins are integrated in the XPLOR-NIH software package. One hundred structures were initially calculated. Upon no NOE violations more than 0.3 Å being found, 30 pairs of hydrogen bond constraints involving slowly exchanging amide protons consistently presented in the initially calculated structures, were introduced as further constraints in the final stage of the structure calculations. Finally, the best 20 structures with the lowest energy selected from 100 calculated structures, were used to represent the three-dimensional structures of the Y67H mutant. The conformers of this bundle show no NOE violation more than 0.3 Å, and have backbone atoms root mean square deviation (RMSD) values of 0.77±0.15 Å, heavy atoms RMSD values of 1.15±0.17 Å with respect to their corresponding mean structures (calculated for residues −5–103 and heme group), respectively. Figure 6 demonstrated the backbone superimposition of 20 structures and ribbon representation of three-dimensional solution structures of the Y67H mutant. The breakdown of experimental constraints per residue was summarized in Figure S3 in File S1. The solution structure infers the secondary structures predicted from NOE pattern mentioned above (Figure S2 in File S1). The entire structure statistics for the 20 conformers were summarized in Table 3, where 84.4% of the residues were located in the most-favored regions of the Ramachandran plot for the Y67H mutant. These data indicate that the solution structure of the Y67H mutant is reasonable.

**Table 3:** Experimental restraints and structural statistics for cyt *c* Y67H variant.

parameters	20 structures of the Y67H variant
***Distance restraints from NOEs***	
Total NOE	1699
Intra-residue (i-j = 0)	642
Sequential (|i-j| = 1)	392
Medium range (1<|i-j|<5)	213
Long range (|i-j|>5)	452
***H-bond pairs restraints***	30
***Structural statistics***	
***r.m.s.d versus the mean structure(Å)***	
All backbone atoms	0.77±0.15
All heavy atoms	1.15±0.17
Backbone atoms (secondary structure)	0.48±0.16
Heavy atoms (secondary structure)	0.93±0.17
***r.m.s.d from the experimental restraints***	
NOE distances (Å)	0.0193±0.00099
***RMSD from idealized geometry***	
Bonds(Å)	0.00167±0.000055
Angles(°)	0.23±0.0072
Impropers(°)	0.36±0.0088
***Ramachandran analysis*** a	
Residues in most favored regions	84.4%
Residues in additionally allowed regions	11.1%
Residues in generously allowed regions	3.3%
Residues in disallowed regions	1.1
***Number of bad contacts/100 residues*** b	0
***Overall G-factor*** b	0.18

a The programs PROCHECK and PROCHECK-NMR were used to check the overall quality of the structure and GLY and Pro are excluded from the Ramachandran analysis.

b For the PROCHECK statistic, less than 10 bad contacts per 100 residues, and an overall G-factor larger than −0.5 are expected for a good quality structure.

## Discussion

### Mutation from Tyr67 to His67 changes hydrogen bond network in heme pocket

**Figure 7:**
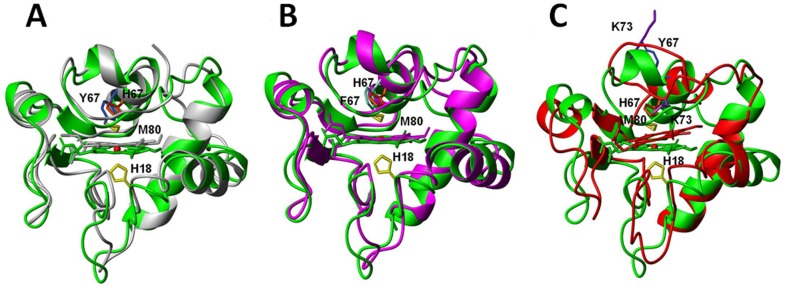
Upon overlaying Cα atoms in second structural region and heme backbone atoms, the conformational comparison: (A) between wild-type cyt *c* (grey, pdb code 1YIC) and its Y67H variant (green); (B) between the Y67F (*magenta*, pdb code 1CTY) and Y67H (green) mutants; (C) between Y67H (green) and alkaline form (red, pdb code 1LMS).

**Figure 8:**
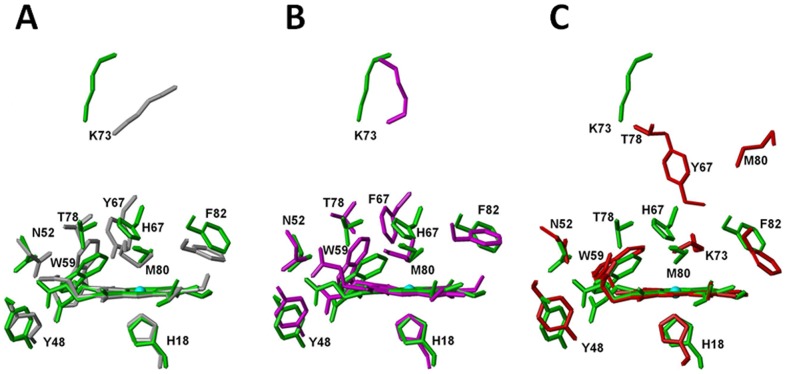
The position comparison of some key residues (including Asn52, Trp59, Tyr67, Lys73, Thr78, Phe82) relative to heme ring upon overlaying heme ring: (A) between the native cyt *c* (*grey*, pdb code 1YIC) and its Y67H variant (*green*); (B) between the cyt *c* Y67H variant (*green*) and the Y67F variant (*magenta*, pdb code 1CTY); (C) between cyt *c* Y67H variant (*green*) and the alkaline form of cyt *c* (*red*, pdb code 1LMS).

To investigate the conformational changes in heme moiety relative to heme ring produced by the mutation from Tyr67 to His67, in Figure 7, the superimpositions were performed by overlaying backbone C*α* atoms in secondary structural regions and heme ring including all carbon and nitrogen atoms to form the π-conjugate porphyrin system. The conformers of native cyt *c* (pdb code 1YIC) and its Y67H mutant are almost identical with a global backbone atoms RMSD value of 1.15 Å, which can explain the fact the exposure of heme group of the Y67H variant to the solvent is quite similar to that of the native form (only 0.1% increment in the Y67H variant evaluated with the program MOLMOL, when considering the iron ion and the carbon and nitrogen atoms to form the π-conjugate porphyrin system.). In native cyt *c*, –OH group of Tyr67 forms one hydrogen bond to Met80 sulfur, holding an internal molecule of water. In the Y67H mutant, although the side-chain of His67 is almost in the same position of Tyr67 side-chain (Figure 8), but the distance between His67 Nε2 and Met80 sulfur is close to 4.0 Å in all final 20 best conformers (obviously larger than that between oxygen atom of Tyr67 –OH and sulfur atom of Met80 is 3.34 Å in the native cyt *c*) (Table S1). Moreover, the distance between His67 Nε2 and heme coordinated Fe^3+^ ion was measured as 5.48 Å, much longer than that (4.30 Å) between the oxygen atom of Tyr67 –OH group and Fe^3+^ ion in the native cyt *c*; the distances in the Y67H mutant between Thr78 Oγ1 atom and oxygen atoms O1α and O2α of heme 6-propionate are 5.11 Å and 6.07 Å, respectively, both larger than those (4.55 Å and 3.44 Å) in the native cyt *c*. The distances between Asn52 Nδ2 and oxygen atoms O1α and O2α of heme 7-propionate were measured as 5.51 Å and 3.87 Å in the Y67H mutant, respectively, also larger than those (2.86 Å and 4.38 Å) in the native cyt *c*. All these differences in distances indicate that the mutation from Tyr67 to His67 disrupts the hydrogen bond between Met80 and residue 67, and also changes the hydrogen bond network. These structural changes weaken the axial Fe-S bond (the Fe-S bond length is 2.19 Å in native cyt *c*, and 2.43 Å in the Y67H mutant), the stability of the Y67H mutant is thus decreased. The weakened Fe-S bond results in slight shift of helix IV (71–74 aa) away from the heme ring (Figure 7A). This accords with the fact that the distance between Lys73 Nζ atom and Fe^3+^ ion is 19.6 Å in the Y67H mutant, close to that (19.0 Å) in the Y67F mutant [18], but clearly longer than that (16.9 Å) in native cyt *c*. Therefore, our NMR structure of the Y67H variant reveals a basis why Y67H is more unstable than its wild-type.

To probe the structural difference between the Y67H and Y67F mutants, we superimposed their conformations (Figure 7B) and measured the distance changes between some key residues (Figure 8B). The Y67H variant has a global backbone atoms RMSD value of 1.49 Å with the Y67F variant (pdb code: 1CTY), implying that their global foldings are almost identical. In comparison, helix IV (71–74 aa) only slightly shifts away from the heme pocket in the Y67H variant, which makes the heme cavity a little looser than that in the Y67F variant. This is confirmed by the following observations. The mutation from Tyr67 to Phe67 destroys the hydrogen bond between Met80 and heme Fe^3+^ (the bond length of Fe-S is 2.44 Å, close to that in the Y67H variant), but the protein stability is increased by forming a more compact conformation than that in native cyt *c* [7]. Moreover, the distances between Trp59 Nε1 and heme 7-propionate carboxyl group O1α atom was measured as 4.46 Å in the Y67H variant, larger than that (3.86 Å, hydrogen bond is observed) in the Y67F variant. The distance between Thr78 Oγ1 atom and the carboxyl atom O2δ in heme 6-propionate is 6.07 Å in the Y67H variant, much larger than that (3.08 Å) in the Y67F mutant. These changes imply that the hydrogen bonds observed in the Y67F variant between Thr78 and heme 6-propionate, and between Trp59 and heme 7-propionate may disappear in the Y67H variant.

As we knew, from the native cyt *c* (*i.e.* the conformer III, pdb code 1YCC or 1YIC) to its alkaline form (also called as conformer IV, pdb code 1LMS) [7], [24]–[26], the conformation of the sixth ligand is switched from Met80 in the native form to Lys73 in the alkaline form, a pH dependent conformational toggling, the ligation between side-chain NH_2_ of Lys73 and heme iron results in the loss of helix IV (71–74 aa). In the Y67H mutant, Met80 is still ligated with Fe^3+^, thus the alkaline form of cyt *c* and the Y67H mutant demonstrate big differences in the conformation with a global backbone atoms RMSD value of 2.64 Å (calculated for residues 9–102 aa), as shown in Figure 7C. The movement of the distal loop in the alkaline form is obviously larger than that in the Y67H mutant, the relative position of helix III (residues 63–69 aa) to the heme ring in Y67H mutant is closer than that in the alkaline form. The relative position between helix II (50–54 aa) and helix III in the Y67H mutant is also closer than that in the alkaline form, implying that the channel from heme pocket to the solvent in the alkaline form is open larger than that in the Y67H variant. This can be confirmed by the distance changes shown in Figure 8C and Table S1. The distances between Tyr48 –OH group and heme 6-propionate O1α and O2α were measured as 5.38 Å and 7.59 Å in the alkaline form, respectively, clearly larger than those (3.46 Å and 5.29 Å) in the Y67H variant. Similarly, the distances between Thr49 –OH group and heme 6-propionate O1α and O2α were measured as 7.26 Å and 8.96 Å in the alkaline form, respectively, much larger than those (3.68 Å and 5.47 Å) in the Y67H variant. Other residues, such as Trp59, Tyr67/His67, Thr78 also shift farther away from heme ring in the alkaline form than those in the Y67H variant. These structural differences once again reveal that cyt *c* possesses functional diversity through conformational variability.

Taken together, the current structure of the Y67H variant interprets why Y67H is the most unstable among native cyt *c*, its Y67H and Y67F mutants, and also the peroxidase activity of the Y67H variant, which will be discussed below.

### Mechanism of cardiolipin peroxidation catalyzed by the Y67H variant

To successfully switch the classical electron transfer protein, cyt *c,* into a peroxidase, firstly we need to understand the main differences on the structural elements between cyt *c* and peroxidase, which were listed below: (1) the 6^th^ axial ligand of heme in peroxidase is vacant for substrate loading; (2) in the heme pocket of peroxidase there is a distal histidine facilitating the formation of compound I intermediate (Figure 3); (3) in the heme pocket of peroxidase the existence of a distal arginine to assist this process. Tyr67 is located at the distal position of heme pocket of the yeast iso-1 cyt *c*, thus, we thought that the mutations from Tyr67 to His67, or to Arg67, might induce a distal histidine or a distal arginine, which could enhance peroxidation activity of cyt *c*. Within our anticipation, in guaiacol peroxidation at pH 7.0 and 16°C, compared to the wild type cyt *c*, the catalytic efficiency *k*_cat_/*K*_m_ for the Y67H variant (where *k*_cat_/*K*_m_ = 89.38 M^−1^s^−1^, *K*_m_ = 0.0096 M, *k*_cat_ = 0.858 s^−1^) is increased by about 16 folds (for wild-type cyt *c*, the value *k*_cat_/*K*_m_ is 5.12 M^−1^s^−1^, *K*_m_ = 0.0127 M, *k*_cat_ = 0.065 s^−1^), while *k*_cat_/*K*_m_ of the Y67R variant is raised by about 8 times (for the Y67R variant, the value *k*_cat_/*K*_m_ is 37.47 M^−1^s^−1^, *K*_m_ = 0.0162 M, *k*_cat_ = 0.607 s^−1^) [16]-[17]. The *k*_cat_/*K*_m_ value for the Y67H variant at 42°C (*k*_cat_/*K*_m_ = 984.83 M^−1^s^−1^, *K*_m_ = 0.0087 M, *k*_cat_ = 8.568 s^−1^) was about 10-fold greater than that at 16°C, while for the Y67R variant, the *k*_cat_/*K*_m_ value (*k*_cat_/*K*_m_ = 1844.44 M^−1^s^−1^, *K*_m_ = 0.0090 M, *k*_cat_ = 16.600 s^−1^) was almost 50-fold higher. In the Y67R variant, the mutation from Tyr67 to Arg67 may produce not only a distal arginine (although its structure is not available), but also a room for substrate coordination (because Fe-S bond is broken in the Y67R variant). Our current NMR structure of the Y67H variant (determined at pH 7.0) suggests that the sixth ligation bond Fe-S is attenuated due to the disruption of hydrogen bond between Met80 and Tyr67 existed in the native cyt *c*. The distance between Nε atom of His67 and heme ferric iron is measured as 5.48 Å in the Y67H variant, very close to the distances of 5.84 Å and 5.55 Å between Nε2 atom of the distal histidine and heme iron ion in horseradish peroxidase (HRP, pdb codes 1H5A) and cytochrome *c* peroxidase (C*c*P, pdb codes 2CYP)[14], [15], respectively. Therefore, in the Y67H variant, His67 may act as a distal histidine to increase the peroxidase activity. These results correlate with the peroxidase activity and conformational changes of cyt *c* resulted by the site-directed mutations under the physiological conditions (neutral pH) and without denaturant for the first time.

However, the Y67H variant has much higher peroxidase activity at pH values from 4.0 to 5.2 than those at any other pH conditions (Figure 4), in contrast, native cyt *c* has very low peroxidase activity at any pH condition. How do we explain this phenomenon? In native cyt *c*, the hydrogen bond network, including the hydrogen bond between Tyr67 and Met80, stabilizes the ligation between Met80 and heme Fe^3+^ ion. Thus, the pH titration neither changes the hydrogen bond network, nor weakens the bond between Met80 sulfur atom and heme Fe^3+^ ion (this analysis is consistent with the results from UV-visible and CD spectroscopy [16]–[17]). While in the Y67H variant, the removal of the hydrogen bond between Tyr67 and Met80 weakens the heme-Met80 ligation, leading to that Met80 easily detaches from heme ring. The cleavage of Fe-S bond in the Y67H variant results in a channel in the heme crevice to H_2_O_2_ and substrate for peroxidation, which is a characteristic of the cardiolipin induced conformational transition in cyt *c*. Therefore, at pH values from 4.0 to 5.2, two following elements may stimulate the Y67H variant with the highest peroxidase activity: 1) His67 acts as a distal histidine; 2) vacancy generation specific for the substrate or H_2_O_2_ loading. When pH is less than 4.0, the peroxidase activity of the Y67H variant is dramatically decreased due to the production of the low-spin intermediate His18-heme-water, confirmed by a sharp increase at the absorbance at 620 nm, comparable to the wild-type cyt *c* [17].

At pH conditions from 4.0 to 5.2 in the absence of H_2_O_2_, the neutral aromatic ring of residue His67 of the Y67H variant is protonated (Figure 3). Thus, the protonated His67 in the Y67H variant directly acts an acid to cleave O-O bond in H_2_O_2_, losing a molecule of water. In contrast, the distal histidine in the peroxidases HRP or CcP is proposed to work as a dual acid-base catalyst in the peroxidation reaction (Figure S4 in File S1) [28], where the distal histidine at first functions as a general base to deprotonate H_2_O_2_, then protonated histidine acts as an acid to cleave O-O bond. By omitting the step to deprotonate H_2_O_2_, the protonation of His67 in the Y67H variant facilitates the peroxidation reaction of H_2_O_2_ with heme Fe^3+^ ion. Figure 3 displays a mechanism about how the compound I is formed through the reaction catalyzed by the Y67H variant at pH 4.0. In the absence of the substrate guaiacol for peroxidation, the compound I reacts with H_2_O_2_, resulting in quick heme ring destruction. Otherwise, the compound I will be directly switched into compound II by reaction with guaiacol, further returning to the resting state, *i.e.,* heme Fe^3+^ ion in the high-spin state.

In summary, to probe the mechanism about how cyt *c* functions as a peroxidase in cell pro-apoptosis, we determined the solution structure of the Y67H variant, and demonstrated the intrinsic differences in the peroxidase activities between native cyt *c* and its Y67H variant. The structure indicates that His67 in the Y67H variant works as a distal histidine, and the sixth ligation bond between heme Fe^3+^ and Met80 sulfur atom is weakened by the disruption of hydrogen bond between Tyr67 and Met80. The weakened Fe-S bond makes it more possible to be cleaved at low pH condition. His67 protonation of the Y67H variant facilitates O-O bond cleavage of H_2_O_2_, which further enhances peroxidase activity of the Y67H variant.

## Materials and Methods

### Protein overexpression and purification and NMR sample preparation

The mutation from Tyr67 to His67 was carried out by a QuikChange site-directed mutagenesis kit (Stratagene Inc). The native cyt *c* and its Y67H mutant were expressed in *E coli* containing the phagemid pBTR2 as described elsewhere [16]–[17]. The purification of the native cyt *c* and its Y67H mutant was performed according to the previously published methods [29]–[31]. The homogeneity of the native and mutant protein was examined by running SDS-PAGE gel electrophoresis and further confirmed by electrospray mass spectrometry. For NMR experiments, about 10 mg of each protein was dissolved in 50 mM aqueous phosphate buffer in 90% H_2_O and 10% D_2_O, and the pH value of the solution was carefully adjusted to 7.0. The final concentration was approximately 3 mM in Shigemi NMR tube, determined by running pyridine hemeochrome spectroscopy [32]–[33]. The sample in D_2_O was prepared by lyophilizing the sample in D_2_O two times and then dissolved in 99.96% D_2_O.

### Reaction of H_2_O_2_ with native cyt *c* and its Y67H mutant

To investigate how heme was destructed by H_2_O_2_ in the absence of guaiacol, the reactions of H_2_O_2_ with native cyt *c* and its Y67H mutant were performed by mixing 2 ml of protein solution (∼2 µM in buffer of 100 mM sodium phosphate, pH 6.0) and 5–10 µl of 1 mM H_2_O_2_ (reacting with Y67H) or 10 mM H_2_O_2_ (reacting with native cyt *c*) solution, and monitored by recording the UV-visible spectrum of native cyt *c* and its Y67H mutant by every 20 seconds at 25°C on a Hewlett-Packard 8453 diode array spectrophotometer. The *k*_obs_ value for heme destruction was set equal to ln(2/T_1/2_), where T_1/2_ was the half life-time for the protein when the absorbance at 410 nm was switched into half of the initial absorbance at 410 nm in the reaction system.

### Steady-state kinetic studies

The steady-state kinetics of oxidation of guaiacol by hydrogen peroxide were studied with a SF-61 DX2 stopped-flow apparatus (Hi-Tech, UK) thermostated at 25±0.1°C. The H_2_O_2_ solution was prepared with 30% stock solution and its final concentration was determined with an absorption coefficient of 39.4 M^−1^cm^−1^ at 240 nM [34]. A solution of the native cyt *c* or its Y67H variant (2.0 µM), guaiacol (100 µM) in 100 mM buffer (phosphate buffer for pH 2.0, 3.0 and pH 6.0–8.0, sodium acetate buffer for pH 4.0–6.0, sodium borate buffer for pH 8.5–10, respectively) and a solution of 200 mM H_2_O_2_ in the same buffer were pre-incubated at 25±0.1°C for 5 min. Then both solutions were mixed together in the mixing cell of the stopped-flow instrument to start the oxidation reaction. The steady-state reaction rates were obtained by monitoring the absorbance increase at 470 nm using a molar absorption coefficient of 26.6 m M^−1^cm^−1^ [35]–[36].

The product formation curve of guaiacol oxidation catalyzed by native cyt *c* or its Y67H mutant, is similar to that catalyzed by *Paracoccus versutus* cytochrome *c*-550 [37]–[38]. The rate of the steady-state reaction was determined by taking the maximum of the first derivative of the product formation curve (*i.e.* the linear phase). The peroxidase activity was represented by the AU_470_/s value, which was the absorbance of the product tetraguaiacol at 470 nm at each pH condition.

### NMR spectroscopy

All NMR experiments were acquired at 20°C on a Varian Unity Inova 600 spectrometer with cryo-probe equipped with three channels and pulse-field gradient (where the corresponding ^1^H frequency is 600.2 MHz), or on a Bruker AVANCE III-800 spectrometer with cryo-probe equipped with four channels and pulse-field gradient, where the corresponding ^1^H frequency is 800.2 MHz. The dipolar connectivities were revealed by 2D ^1^H-^1^H NOESY experiments. To detect connectivities among hyperfine-shifted signals, the 2D ^1^H-^1^H NOESY spectra with a spectral width of 76 ppm in both frequency dimensions [39], with a recycle time of 150 ms and a mixing time of 45 ms, were acquired. To confirm assignment of heme protons, the 1D NOE experiments were performed with the superWEFT pulse sequence and the data connected by using standard methodology [40]. To optimize the detection of connectivities in the diamagnetic region (from −2 ppm to 11.5 ppm), ^1^H-^1^H NOESY spectra were acquired with a recycle time of 1.5 s and mixing times of 50, 100, 150, and 200 ms, respectively. ^1^H-^1^H TOCSY spectra were obtained using the spin-lock times of 30, 50, and 80 ms in H_2_O and D_2_O [41], respectively. ^1^H-^1^H double-quantum filtered COSY (DQF-COSY) spectra were recorded in H_2_O and D_2_O [42]. WATERGATE pulse sequence [43] was used for water signal suppression in NOESY spectra in H_2_O, while in other cases pre-saturation was used. All data consisted of 4K data points in the acquisition dimension and 1K points in the indirect dimension. Raw data were weighted with a squared cosine function, zero-filled, and Fourier-transformed to obtain a final matrix 4096×4096 data points. All spectra were collected at 20°C either on the H_2_O or on the D_2_O samples, processed by NMRPipe [44] and analyzed by Sparky 3 (http://www.cgl.ucsf.edu/home/sparky/).

### NMR solution structure calculations

The solution structure calculations were carried out using a standard simulated annealing protocol implemented in the program XPLOR 2.19 (NIH version) [27]. The inter-proton distance restraints derived from NOE intensities were grouped into three distance ranges 1.8–2.9 Å, 1.8–3.5 Å and 1.8–6.0 Å, corresponding to strong, medium and weak NOEs, respectively. Hydrogen bond constraints were introduced based on the un-exchanged backbone amide protons identified in DQF-COSY acquired in D_2_O and the assigned inerratic NOE patterns belonging to the α-helix conformation [45] (Figure S2 in File S1). The default values for the bond and angle force constants in X-PLOR were employed (500 kcal/mol Å^−2^ and 70 kcal/mol Å^−2^, respectively). A total of 100 structures were calculated, and finally, 20 structures with the lowest energy had no NOE violation >0.3 Å. Structure statistics for these 20 structures were summarized in Table 3. The programs PROCHECK [46] and PROCHECK-NMR [47] were used to evaluate overall quality of the calculated solution structures.

### Data deposition

The coordinates of the solution structure of Y67H mutant had been deposited with RCSB Protein Data Bank under accession number 2mhm and ID code rcsb103626, and the assignments of its NMR signals had been deposited with BMRB (Biological magnetic resonance data bank) under accession number 19638, respectively.

## Supporting Information

Table S1 The measured distances between some key residues and heme ring in different states of cyt *c* including native cyt *c*, its Y67H and Y67F mutants, and the alkaline state of cyt *c*.
(DOCX)

File S1 Supporting figures. Figure S1, NOE patterns involving the heme and its axial ligands of cyt c Y67H mutant. Figure S2, Schematic representation of the sequential and medium-range NOE connectivities involving HN, Hα, and Hβ for cyt c Y67H mutant. Figure S3, The number of experimental NOEs per residues (A) and is correlated with global (black) and local (red) backbone RMSD values per residue (B) calculated from the 20 structures of the lowest-energy family with respect to the average structure of cyt *c* Y67H mutant. Figure S4, Proposed mechanism of compound I formation during guaiacol peroxidation by HRP or CcP. The distal histidine first functions as a general base to deprotonate hydrogen peroxide, and then the protonated hisidine as a acid facilitates the O-O bond cleavage.
(DOCX)

## References

[1] Tokuriki N, Tawfik DS (2009) Protein dynamism and evolvability. Science 324: 203–207. 19359577 10.1126/science.1169375

[2] Henzler-Wildman K, Kern D (2007) Dynamic personalities of proteins. Nature 450: 964–972. 18075575 10.1038/nature06522

[3] Vinson VJ (2009) Proteins in Motion. Science 324: 197. 19359575 10.1126/science.324.5924.197

[4] Ow YP, Green DR, Hao Z, Mak TW (2008) Cytochrome c: functions beyond respiration. Nat Rev Mol Cell Biol 9: 532–542. 18568041 10.1038/nrm2434

[5] Wilson MT, Greenwood C (1996) Cytochrome c: A Multidisciplinary Approach, University Science Books, Sausilito, CA, 611–634p;

[6] Rosell FI, Ferrer JC, Mauk AG (1998) Proton-Linked Protein Conformational Switching: Definition of the Alkaline Conformational Transition of Yeast Iso-1-ferricytochrome c. J Am Chem Soc 120: 11234–11245.

[7] Assfalg M, Bertini I, Dolfi A, Turano P, Mauk G, et al (2003) Structural model for an alkaline form of ferricytochrome c. J Am Chem Soc 125: 2913–2922. 12617658 10.1021/ja027180s

[8] Kagan VE, Tyurin VA, Jiang JF, Tyurina YY, Ritov VB, et al (2005) Cytochrome c acts as a cardiolipin oxygenase required for release of proapoptotic factors. Nat Chem Biol 1: 223–232. 16408039 10.1038/nchembio727

[9] Belikova NA, Vladimirov YA, Osipov AN, Kapralov AA, Tyurin VA, et al (2006) Peroxidase activity and structural transitions of cytochrome c bound to cardiolipin-containing membranes. Biochemistry 45: 4998–5009. 16605268 10.1021/bi0525573 PMC2527545

[10] Kagan VE, Bayir HA, Belikova NA, Kapralov O, Tyurina YY, et al (2009) Cytochrome c/cardiolipin relations in mitochondria: a kiss of death. Free Radical Biol Med 46: 1439–1453. 19285551 10.1016/j.freeradbiomed.2009.03.004 PMC2732771

[11] Pettigrew GW, Leaver JL, Meyer TE, Ryle AP (1975) Purification, properties and amino acid sequence of atypical cytochrome c from two protozoa, *Euglena gracilis* and *Crithidia oncopelti* . Biochem J 147: 291–302. 170910 10.1042/bj1470291 PMC1165443

[12] Takano T, Dickerson RE (1981) Conformation change of cytochrome c. I. Ferrocytochrome c structure refined at 1.5 A resolution. J Mol Biol 153: 79–94. 6279867 10.1016/0022-2836(81)90528-3

[13] Takano T, Dickerson RE (1981) Conformation change of cytochrome c. II. Ferricytochrome c refinement at 1.8 A and comparison with the ferrocytochrome structure. J Mol Biol 153: 95–115. 6279868 10.1016/0022-2836(81)90529-5

[14] Berglund GI, Carlsson GH, Smith AT, Szöke H, Henriksen A, et al (2002) The catalytic pathway of horseradish peroxidase at high resolution. Nature 417: 463–468. 12024218 10.1038/417463a

[15] Finzel BC, Poulos TL, Kraut J (1984) Crystal structure of yeast cytochrome c peroxidase refined at 1.7-A resolution. J Biol Chem 259: 13027–13036. 6092361

[16] Ying T, Wang ZH, Lin YW, Xie J, Tan X, et al (2009) Tyrosine-67 in cytochrome c is a possible apoptotic trigger controlled by hydrogen bonds via a conformational transition. Chem Commun 30: 4512–4514. 10.1039/b904347k 19617967

[17] Ying T, Wang ZH, Zhong F, Tan X, et al (2010) Distinct mechanisms for the pro-apoptotic conformational transition and alkaline transition in cytochrome *c* . Chem Commun 46: 3541–3543. 10.1039/b926261j 20379610

[18] Berghuis AM, Guillemette JG, Smith M, Brayer GD (1994) Mutation of tyrosine-67 to phenylalanine in cytochrome c significantly alters the local heme environment. J Mol Biol 235: 1326–1341. 8308895 10.1006/jmbi.1994.1086

[19] Wuthrich K (1986), NMR of proteins and nucleic acids, John Wiley & Sons, 130–161p.

[20] Cutler RL, Pielak GJ, Mauk AG, Smith M (1987) Replacement of cysteine-107 of *Saccharomyces cerevisiae* iso-1-cytochrome *c* with threonine: improved stability of the mutant protein. Protein Engin 1: 95–99. 10.1093/protein/1.2.95 2853358

[21] Gao Y, Boyd J, Williams RJ, Pielak GJ (1990) Assignment of proton resonances, identification of secondary structural elements, and analysis of backbone chemical shifts for the C102T variant of yeast iso-1-cytochrome *c* and horse cytochrome *c* . Biochemistry 29: 6994–7003. 2171638 10.1021/bi00482a007

[22] Villegas JA, Mauk AG, Vazquez-Duhalt R (2000) A cytochrome c variant resistant to heme degradation by hydrogen peroxide. Chem Biol 7: 237–244. 10780923 10.1016/s1074-5521(00)00098-3

[23] Liu Z, Lin H, Ye S, Liu QY, Meng Z, et al (2006) Remarkably high activities of testicular cytochrome c in destroying reactive oxygen species and in triggering apoptosis. Proc Natl Acad Sci USA 103: 8965–8970. 16757556 10.1073/pnas.0603327103 PMC1482549

[24] Pielak GJ, Boyd J, Moore GR, Williams RJ (1988) Two-dimensional NMR as a probe of structural similarity applied to mutants of cytochrome *c* . Eur J Biochem 177: 167–177. 2846295 10.1111/j.1432-1033.1988.tb14360.x

[25] Banci L, Bertini I, Bren KL, Gray HB, Sompornpisut P, et al (1997) Solution structure of oxidized *Saccharomyces cerevisiae* iso-1-cytochrome *c* . Biochemistry 36: 8992–9001. 9220987 10.1021/bi963025c

[26] Baistrocchi P, Banci L, Bertini I, Turano P (1996) Three-dimensional solution structure of *Saccharomyces cerevisiae* reduced iso-1-cytochrome *c* . Biochemistry 35: 13788–13796. 8901521 10.1021/bi961110e

[27] Kuszewski J, Clore GM (2000) Sources of and solutions to problems in the refinement of protein NMR structures against torsion angle potentials of mean force. J Magn Reson 146: 249–254. 11001840 10.1006/jmre.2000.2142

[28] Ozaki S, Matsui T, Roach MP, Watanabe Y (2000) Rational molecular design of a catalytic site: engineering of catalytic functions to the myoglobin active site framework. Coord Chem Rev 198: 39–59.

[29] Pollock WB, Rosell FT, Twitchett MB, Dumont ME, Mauk AG (1998) Bacterial expression of a mitochondrial cytochrome *c*. Trimethylation of Lys72 in Yeast *iso*-1-Cytochrome *c* and the alkaline conformational transition. Biochemistry 37: 6124–6131. 9558351 10.1021/bi972188d

[30] Rosell FI, Mauk AG (2002) Spectroscopic properties of a mitochondrial Cytochrome *c* with a single thioether bond to the heme prosthetic group. Biochemistry 41: 7811–8. 12056913 10.1021/bi016060e

[31] Silkstone G, Stanway G, Brzezinski P, Wilson MT (2002) Production and characterisation of Met80X mutants of yeast iso-1-cytochrome *c*: spectral, photochemical and binding studies on the ferrous derivatives. Biophys Chem 98: 65–77. 12128190 10.1016/s0301-4622(02)00085-6

[32] Berry EA, Trumpower BL (1987) Simultaneous determination of hemes a, b, and c from pyridine hemochrome spectra. Anal Biochem 161: 1–15. 3578775 10.1016/0003-2697(87)90643-9

[33] Lan WX, Wang Z, Yang Z, Cao C. Huang ZX, et al. (2011) Conformational toggling of Yeast Iso-1-cytochrome c in the oxidized and reduced states, PLoS One 6, e27219. 10.1371/journal.pone.0027219 PMC3210782 22087268

[34] Nelson DP, Kiesow LA (1972) Enthalpy of decomposition of hydrogen peroxide by catalase at 25 degrees C (with molar extinction coefficients of H_2_O_2_ solutions in the UV). Anal Biochem 49: 474–478. 5082943 10.1016/0003-2697(72)90451-4

[35] Baldwin DA, Marques HM, Pratt JM (1987) Hemes and hemoproteins. 5: Kinetics of the peroxidatic activity of microperoxidase-8: model for the peroxidase enzymes. J Inorg Biochem 30: 203–217. 2821191 10.1016/0162-0134(87)80064-8

[36] DePillis GD, Sishta BP, Mauk AG, Ortiz de Montellano PR (1991) Small substrates and cytochrome c are oxidized at different sites of cytochrome c peroxidase. J Biol Chem 266: 19334–19341. 1655784

[37] Diederix RE, Ubbink M, Canters GW (2001) The peroxidase activity of cytochrome c-550 from Paracoccus versutus. Eur J Biochem 268: 4207–4216. 11488914 10.1046/j.1432-1327.2001.02335.x

[38] Diederix RE, Fittipaldi M, Worrall JA, Hubber M, Ubbink M, et al (2003) Kinetic stability of the peroxidase activity of unfolded cytochrome c: heme degradation and catalyst inactivation by hydrogen peroxide,. Inorg Chem 42: 7249–7257. 14577794 10.1021/ic0343861

[39] Macura S, Wuthrich K, Ernst RR (1982) The relevance of J cross-peaks in two-dimensional NOE experiments of macromolecules. J Magn Reson 47: 351–357.

[40] Banci L, Bertini I, Luchinat C, Piccioli M, Scozzafava A, et al (1989) Proton NOE studies on dicopper(II) dicobalt(II) superoxide dismutase. Inorg Chem 28: 4650–4656.

[41] Bax A, Davis DG (1985) MLEV-17-based two-dimensional homonuclear magnetization transfer spectroscopy. J Magn Reson 65: 355–360.

[42] Silkstone GG, Cooper CE, Svistunenko D, Wilson MT (2005) EPR and optical spectroscopic studies of Met80X mutants of yeast ferricytochrome *c*. Models for Intermediates in the alkaline transition. J Am Chem Soc 127: 92–99. 15631458 10.1021/ja045719b

[43] Piotto M, Saudek V, Sklenar V (1992) Gradient-tailored excitation for single-quantum NMR spectroscopy of aqueous solutions. J Biomol NMR 2: 661–665. 1490109 10.1007/BF02192855

[44] Delaglio F, Grzesiek S, Vuister GW, Zhu G, Pfeifer J, et al (1995) NMRPipe: a multidimensional spectral processing system based on UNIX pipes. J Biomol NMR 6: 277–293. 8520220 10.1007/BF00197809

[45] Murphy ME (1993) Ph.D. dissertation, University of British Columbia, 96–112p.

[46] Laskowski RA, MacArthur MW, Moss DS, Thornton JM (1993) PROCHECK: a program to check the stereochemical quality of protein structures. J. Appl Cryst 26: 283–291.

[47] Laskowski RA, Rullmann JAC, MacArthur MW, Kaptein R, Thornton JM (1996) AQUA and PROCHECK-NMR Programs for checking the quality of protein structures solved by NMR,. J Biomol NMR 8: 477–486. 9008363 10.1007/BF00228148

[48] Koradi R, Billeter M, Wuthrich K (1996), MOLMOL a program for display and analysis of macromolecular structures, J Mol Graph 14: 51–55, 29–32. 10.1016/0263-7855(96)00009-4 8744573

