# Supplementary material for: Structural Basis for Cytochrome c Y67H Mutant to Function as a Peroxidase
Source: PLoS One. 2014 Sep 11;9(9):e107305. doi: 10.1371/journal.pone.0107305 (PMC4161393; doi:10.1371/journal.pone.0107305)
Supplement: Table S1 — The measured distances between some key residues and heme ring in different states of cyt c including native cyt c, its Y67H and Y67F mutants, and the alkaline state of cyt c. (DOCX) [file pone.0107305.s001.docx]

**Table S1** The measured distances between some key residues and heme ring in different states of cyt *c* including native cyt *c*, its Y67H and Y67F mutants, and the alkaline state of cyt *c*.

|  | Wild-type | Y67F | Y67H | Alkaline_oxi |
| --- | --- | --- | --- | --- |
| His18(NE2)-Fe | 1.85 | 2.05 | 2.10 | 1.96 |
| Tyr48(OH)-HEC(O1A) | 2.61 | 3.50 | 3.46 | 5.38 |
| Tyr48(OH)-HEC(O2A) | 4.43 | 4.80 | 5.29 | 7.59 |
| Thr49(OG1)-HEC(O1D) | 2.62 | 4.00 | 3.68 | 7.26 |
| Thr49(OG1)-HEC(O2D) | 4.14 | 2.71 | 5.47 | 8.96 |
| Asn52(ND2)-HEC(O1A) | 2.86 | 6.03 | 5.51 | 4.43 |
| Asn52(ND2)-HEC(O2A) | 4.38 | 4.77 | 3.87 | 4.00 |
| Trp59(NE1)-HEC(O1A) | 2.93 | 3.86 | 4.46 | 5.76 |
| Trp59(NE1)-HEC(O2A) | 4.16 | 2.89 | 2.56 | 5.17 |
| Trp59(NE1)-Fe | 9.20 | 9.42 | 9.32 | 9.15 |
| Tyr67(OH)-Fe | 4.30 |  |  | 15.22 |
| His67(NE2)-Fe |  |  | 5.48 |  |
| Phe67(CZ)-Fe |  | 5.59 |  |  |
| Tyr67(OH)-Met80(SD) | 3.34 |  |  | 13.54 |
| His67(NE2)-Met80(SD) |  |  | 3.9 |  |
| Phe67(CZ)-Met80(SD) |  | 4.20 |  |  |
| Lys73(NZ)-Fe | 16.93 | 19.00 | 19.60 | 2.04 |
| Thr78(OG1)-HEC(O1D) | 4.55 | 4.92 | 5.11 | 12.70 |
| Thr78(OG1)-HEC(O2D) | 3.55 | 3.08 | 6.07 | 14.74 |
| Met80(SD)-Fe | 2.19 | 2.44 | 2.43 | 15.16 |
| Phe82(CB)-Fe | 5.34 | 6.06 | 6.54 | 7.15 |
| Phe82(CZ)-Fe | 7.30 | 8.59 | 8.50 | 8.62 |
