# Supplementary material for: Structural Basis for Cytochrome c Y67H Mutant to Function as a Peroxidase
Source: PLoS One. 2014 Sep 11;9(9):e107305. doi: 10.1371/journal.pone.0107305 (PMC4161393; doi:10.1371/journal.pone.0107305)

Structural basis for cytochrome *c* to function as a peroxidase

Wenxian Lan^a§^, Zhonghua Wang^b§^, Zhongzheng Yang^a^, Tianlei Yingb^b^, Xu Zhang^c^, Xiangshi Tan^b^, Maili Liu^c^, Chunyang Cao^a^*, Zhong-Xian Huang^b^*

**Supplementary data, figure and table legends**

**Figure S1** NOE patterns involving the heme and its axial ligands of cyt *c* Y67H mutant.


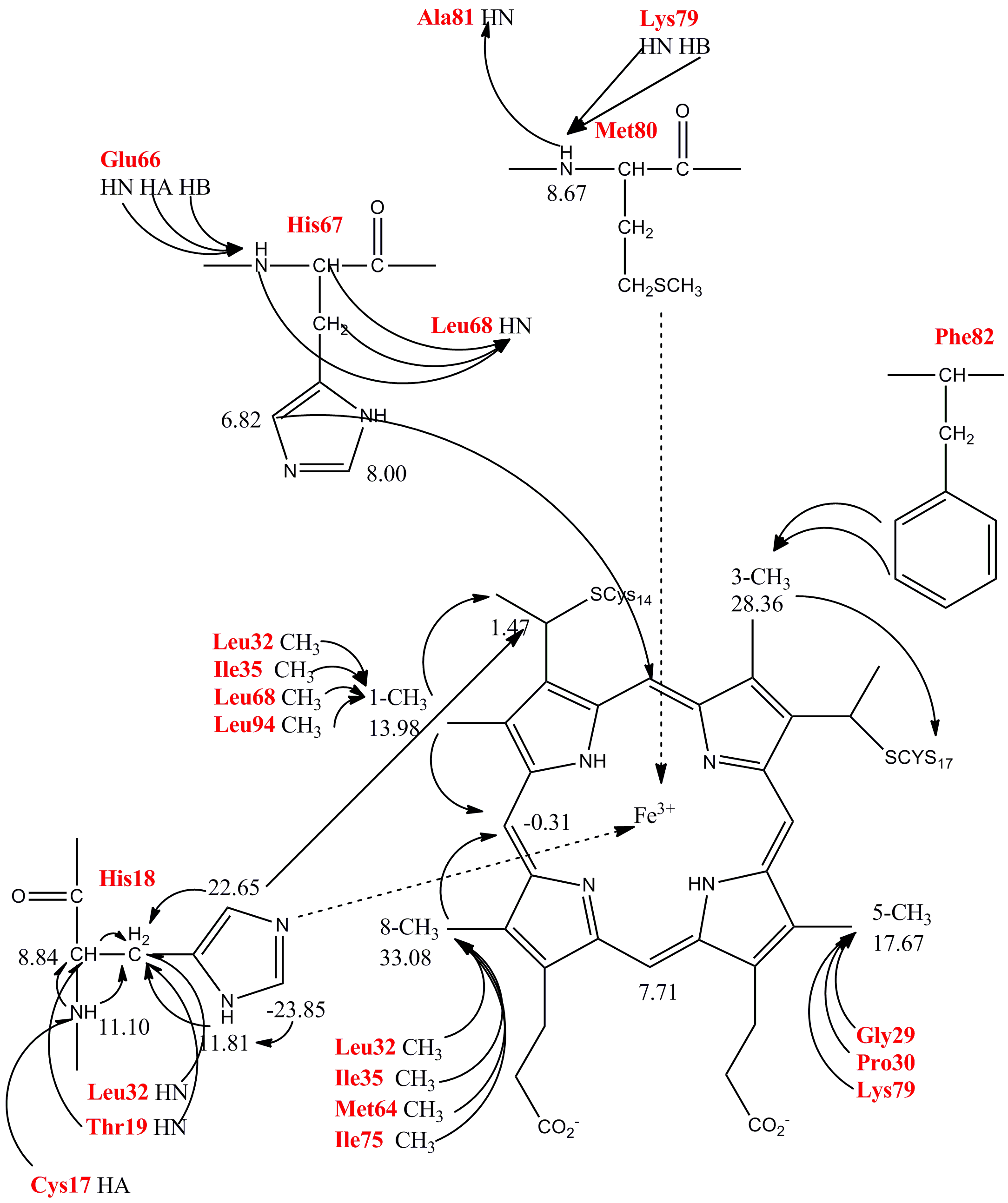


Figure S2 Schematic representation of the sequential and medium-range NOE connectivities involving HN, Hα, and Hβ for cyt *c* Y67H mutant.


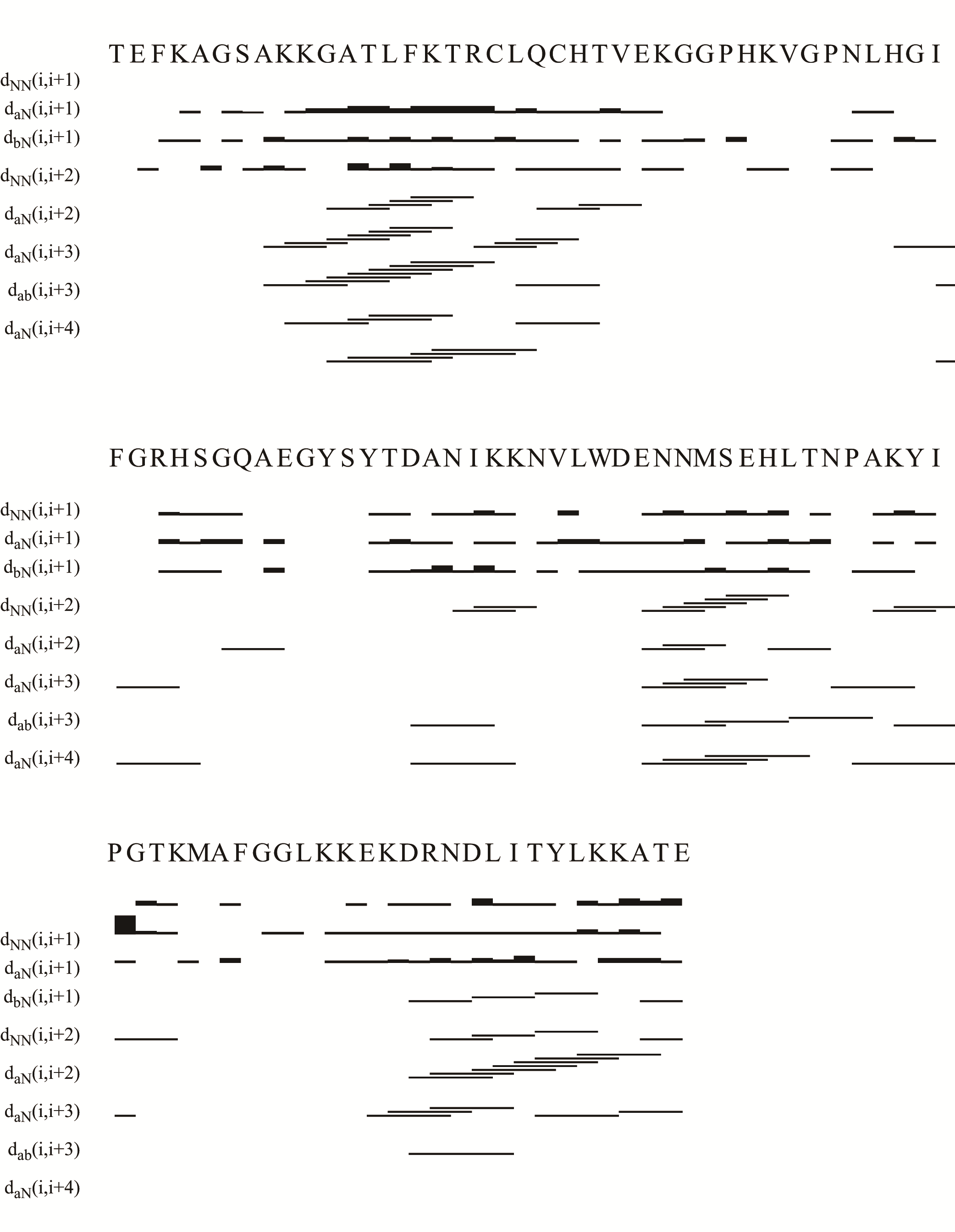


Figure S3 The number of experimental NOEs per residues (A) and is correlated with global (black) and local (red) backbone RMSD values per residue (B) calculated from the 20 structures of the lowest-energy family with respect to the average structure of cyt *c* Y67H mutant.

Figure S4 Proposed mechanism of compound I formation during guaiacol peroxidation by HRP or CcP . The distal histidine first functions as a general base to deprotonate hydrogen peroxide, and then the protonated hisidine as a acid facilitates the O-O bond cleavage.


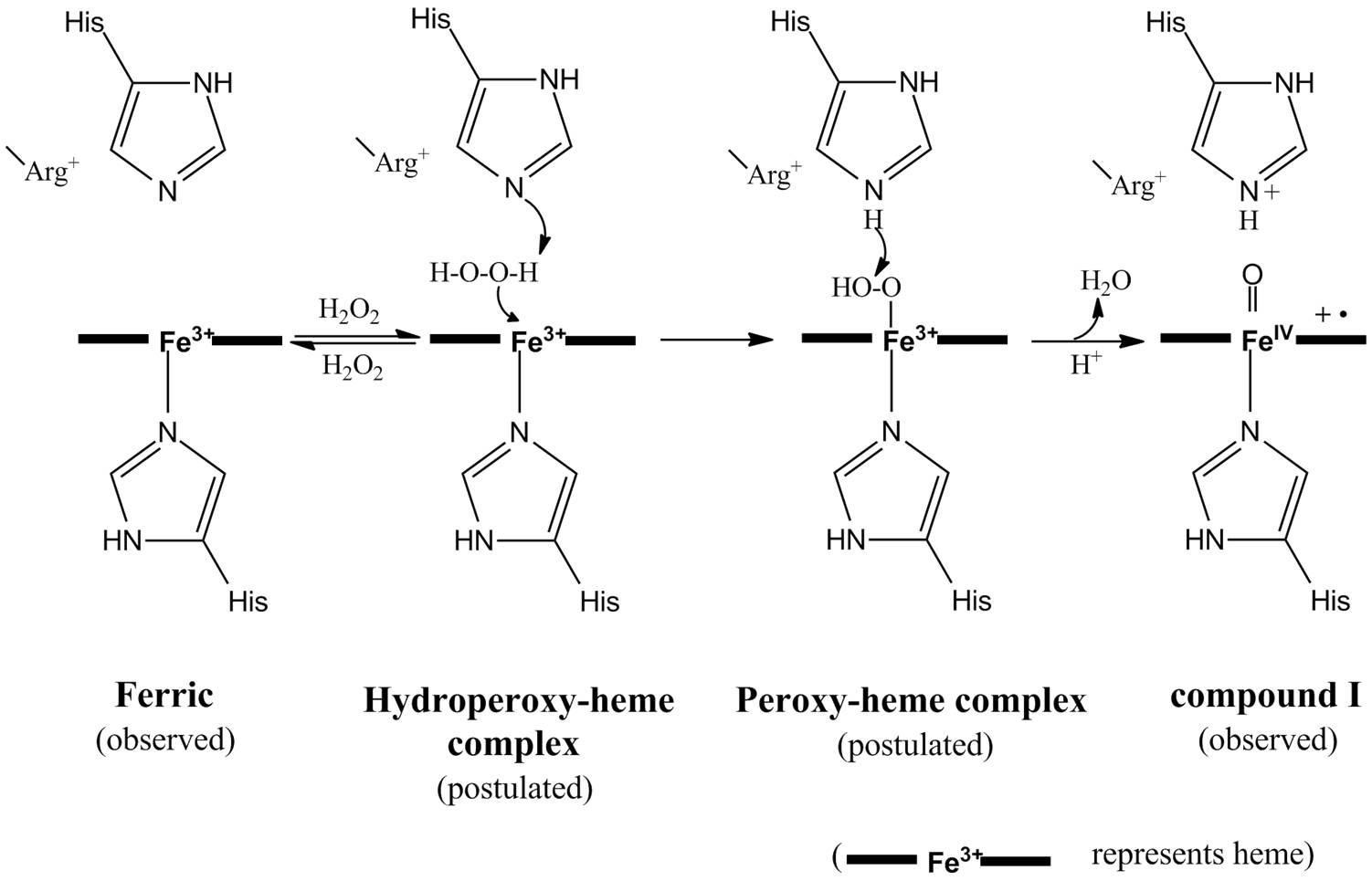

Supplement: File S1 — Supporting figures. Figure S1, NOE patterns involving the heme and its axial ligands of cyt c Y67H mutant. Figure S2, Schematic representation of the sequential and medium-range NOE connectivities involving HN, Hα, and Hβ for cyt c Y67H mutant. Figure S3, The number of experimental NOEs per residues (A) and is correlated with global (black) and local (red) backbone RMSD values per residue (B) calculated from the 20 structures of the lowest-energy family with respect to the average structure of cyt c Y67H mutant. Figure S4, Proposed mechanism of compound I formation during guaiacol peroxidation by HRP or CcP. The distal histidine first functions as a general base to deprotonate hydrogen peroxide, and then the protonated hisidine as a acid facilitates the O-O bond cleavage. (DOCX) [file pone.0107305.s002.docx]
